# *MET* and *NF2* alterations confer primary and early resistance to first‐line alectinib treatment in *ALK*‐positive non‐small‐cell lung cancer

**DOI:** 10.1002/1878-0261.70029

**Published:** 2025-04-01

**Authors:** Jie Hu, Ning Ding, Xiaobo Xu, Yedan Chen, Yong Zhang, Jingwen Liu, Jiebai Zhou, Hairong Bao, Donghui Zhang, Yijun Song, Yang Shao, Yuanlin Song

**Affiliations:** ^1^ Department of Pulmonary Medicine Shanghai Geriatric Medical Center China; ^2^ Department of Pulmonary Medicine, Zhongshan Hospital Fudan University Shanghai China; ^3^ Shanghai Respiratory Research Institute China; ^4^ Geneseeq Research Institute Nanjing Geneseeq Technology Inc. China; ^5^ School of Public Health Nanjing Medical University China; ^6^ Shanghai Key Laboratory of Lung Inflammation and Injury, Department of Pulmonary Medicine, Zhongshan Hospital Fudan University Shanghai China

**Keywords:** alectinib resistance, *ALK*‐rearranged NSCLC, *EML4‐ALK* variants, *MET*, *NF2*

## Abstract

Although first‐line alectinib has prolonged survival in *ALK‐*mutated non‐small‐cell lung cancers (NSCLCs), the response to treatment varies among patients, and the primary/early development of alectinib resistance mechanisms is still not fully understood. Here, we analyzed molecular profiles of 108 alectinib‐treated patients (first‐line and second‐line after crizotinib) with confirmed relapse by targeted sequencing of cancer‐related genes. After first‐line treatment, off‐target *MET* and *NF2* alterations were more frequent than on‐target alterations within the first 6 months, causing primary or early resistance. Conversely, on‐target alterations became prevalent after 1 year of first‐line alectinib treatment and predominantly after second‐line. The incidence of acquired resistance also depended on *EML4‐ALK* variants. In variant 1 (v1), off‐target alterations were responsible for 50% of resistance cases after first‐line alectinib therapy, whereas on‐target mutations had no contribution in this subgroup. In variant 3 (v3), on‐target alterations resulted in 46% of resistance cases, whereas only 18% were caused by off‐target mutations. After second‐line treatment, the most common mutations in v1 were L1196M (42%) and G1269A (25%), while G1202R was detected in 45% of v3 tumors. These findings emphasize the importance of stratifying resistance mechanisms to guide tailored treatment for *ALK*‐positive NSCLCs.

## Graphical abstract

Alectinib resistance in *ALK*+ NSCLC depends on treatment sequence and *EML4‐ALK* variants. Variant 1 exhibited off‐target resistance after first‐line treatment, while variant 3 and later lines favored on‐target mutations. Early resistance involved off‐target alterations, like *MET* and *NF2*, while on‐target mutations emerged with prolonged therapy. Our findings provided important insights into variant‐specific strategies for the precision management of *ALK*‐driven NSCLC.

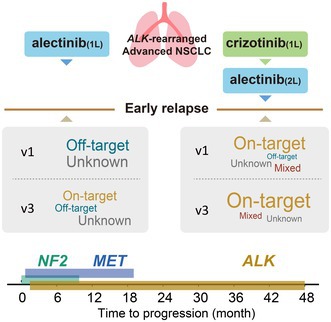

**Abbreviations**
ALK: anaplastic lymphoma kinase
BRAF: V‐Raf murine sarcoma viral oncogene homolog B
CNV: copy number variation
EGFR: epidermal growth factor receptor
EML4: echinoderm microtubule‐associated protein‐like 4
EMT: epithelial‐to‐mesenchymal transition
ERBB2: Erb‐B2 receptor tyrosine kinase 2
FFPE: formalin‐fixed paraffin‐embedded
FGFR3: fibroblast growth factor receptor 3
HER3: human epidermal growth factor receptor 3
HR: hazard ratio
HRAS: Harvey rat sarcoma viral oncogene homolog
IGF1R: insulin‐like growth factor‐1 receptor
KDD: kinase domain duplication
KDM: kinase domain mutation
KRAS: Kirsten rat sarcoma viral oncogene homolog
MET: mesenchymal–epithelial transition
NF2: neurofibromin 2
NGS: next‐generation sequencing
NRAS: neuroblastoma RAS viral oncogene homolog
NSCLC: non‐small‐cell lung cancer
PFS: progression‐free survival
PIK3CA: phosphatidylinositol‐4,5‐bisphosphate 3‐kinase catalytic subunit alpha
PTEN: phosphatase and tensin homolog
ROS1: ROS Proto‐Oncogene 1
SNP: single‐nucleotide polymorphism
SNV: single nucleotide variations
TAPE: tandem atypical propeller EML
TKI: tyrosine kinase inhibitor
TP53: tumor protein P53
VAF: variant allele frequency

## 1 Introduction

Approximately 3–7% of non‐small‐cell lung cancers (NSCLC) are caused by rearrangements in the anaplastic lymphoma kinase (*ALK*) gene [1]. These rearrangements lead to constitutive activation of ALK signaling, which promotes cell proliferation and tumor growth. In the last decade, the development and approval of ALK tyrosine kinase inhibitors (TKIs) have revolutionized the therapeutic landscape for advanced *ALK*‐positive NSCLC. Alectinib is a highly selective and potent second‐generation ALK TKI. It has shown remarkable efficacy in both naïve and crizotinib‐pretreated *ALK*‐fusion positive NSCLCs [2, 3, 4, 5]. With a favorable progression‐free survival (PFS) of 34.8 months with alectinib versus 10.9 months with crizotinib in untreated patients [4], alectinib has been approved and recommended as a first‐line regimen for *ALK*‐positive metastatic NSCLC. Echinoderm microtubule‐associated protein‐like 4 (EML4) is the predominant fusion partner, representing approximately 85% of all *ALK* fusions [6]. Based on the chromosomal location of the breakpoints, several different *EML4‐ALK* variants have been identified, resulting in a range of fusion protein forms. Notably, studies have shown that different *EML4‐ALK* variants display differential responses to ALK TKIs, particularly between the two most common variants, variant 1 and variant 3 [7, 8]. This raises the question of whether distinct *ALK* variants are also associated with different resistance mechanisms in patients experiencing early relapse.

Like all targeted therapies, after initial responsiveness to ALK TKIs, cells ultimately evolve to become resistant, leading to disease relapse. Approximately 30% of first‐line alectinib‐treated patients became refractory within 1 year of treatment due to various mechanisms of secondary or acquired resistance [2]. Numerous studies have revealed that acquired ALK TKI resistance can result from on‐target alterations, including point mutations in the tyrosine kinase domain of *ALK* and *ALK* amplification, which restore the *ALK* kinase's activity [9]. These on‐target alterations are prevalent in 50–70% of patients after second‐generation ALK TKIs, with G1202R being the most common mutation [10, 11, 12]. Another major cause of resistance is due to off‐target mutations that bypass the inhibitory effects. *MET* alterations, particularly *MET* amplification and exon 14 skipping mutations, are the main recurring off‐target mechanisms causing ALK TKI resistance [13, 14]. Other than *MET*, *in vitro* studies also showed that alternative activation of tyrosine kinase receptors could confer resistance to alectinib through increased activities of insulin‐like growth factor‐1 receptor (IGF1R) and human epidermal growth factor receptor 3 (HER3) [15].

*MET* alterations are also more common in patients treated with second‐generation TKIs (ceritinib, brigatinib, or alectinib) than in those receiving crizotinib as upfront therapy [16], which implies potential differences in resistance landscapes depending on treatment sequence. Besides acquired resistance, primary or intrinsic resistance to ALK TKIs is less understood. Primary resistance occurs when patients fail to achieve an objective response and have progressive disease within a short period of time, typically within 3 months of treatment [17]. For *ALK*‐rearranged patients, approximately 20–25% treated with first‐line crizotinib and 8–17% treated with first‐line alectinib experienced primary resistance, but the mechanisms behind this are unclear [3, 11, 18]. Unlike EGFR‐positive NSCLCs, *de novo ALK* kinase domain mutations (KDM) are rarely found in TKI‐naïve *ALK*‐rearranged patients. Furthermore, there is no established proof that preexisting activated bypass signaling causes intrinsic resistance to ALK TKIs.

Here, we analyze mutational profiles of tumor tissue or liquid biopsy samples from real‐world *ALK*‐positive NSCLC patients who had relapsed on first‐ or second‐line alectinib treatment to elucidate potential resistance mechanisms. Of note, by the data cut‐off date, October 2020, alectinib had only been approved in China for 2 years (since August 2018). Therefore, most patients included in this study did not achieve the expected survival benefit from the drug due to primary or early development of resistance. Our work stratifies the distinct resistance mechanisms associated with lines of treatment and *ALK* fusion subtypes, which might offer valuable insights for future therapeutic decisions.

## 2 Materials and methods

### 2.1 Patient samples and clinical efficacy assessment

We retrospectively screened 120 patient records with ALK‐fusion positive advanced NSCLC diagnosed between January 2014 and October 2020. A total of 108 patients with detectable genetic alterations by next‐generation sequencing (NGS) were included in this study. Of these, 52 received alectinib as a first‐line regimen (first‐line cohort) and 56 received second‐line alectinib following crizotinib (second‐line cohort). In the first‐line cohort, 27 patients had paired pre‐ and postalectinib biopsies. Additionally, the second‐line cohort included 11 paired biopsies before and after alectinib treatment (Fig. S1).

Genomic profiles of pre‐ and postalectinib biopsies were compared. Types of biopsies tested included tissue, plasma, cerebrospinal fluid, and pleural effusion. To overcome tumor heterogeneity, we pooled genetic alterations detected in all types of qualified biopsies collected at the same timepoint for analyses. All samples were matched with whole blood samples as negative controls. PFS for first‐ or second‐line treatments was defined as the time from initiation to disease progression or last follow‐up. This study was approved by the Ethics Committee of Zhongshan Hospital, Fudan University (Approval No: B2017‐142R), and the methods used in this study were in accordance with the Declaration of Helsinki. The samples were collected at Zhongshan Hospital, Fudan University. Written informed consents were obtained from all the participants for genetic testing and research.

### 2.2 Library preparation and sequencing

Formalin‐fixed paraffin‐embedded (FFPE) sections and liquid biopsy samples were sent to the CAP/CLIA (College of American Pathologists and Clinical Laboratory Improvements Amendments) accredited central laboratory at Nanjing Geneseeq Technology Inc. (Nanjing, China) for genomic DNA extraction and hybridization capture based NGS targeting 425 or 139 cancer‐relevant genes. Library preparation was performed following previously described protocols [19]. Five to 10 formalin‐fixed paraffin‐embedded (FFPE) sections from each sample were used to extract genomic DNA using the QIAamp DNA FFPE Tissue Kit (Qiagen, Germany). Extracted DNA was quantified by the Qubit dsDNA HS Assay Kit (Thermo Fisher Scientific, Waltham, MA, USA) and its purity was measured by NanoDrop 2000 Spectrophotometers (Thermo Fisher Scientific). DNA was then fragmented to a size around 350 bp by using the Covaris M220 sonication system (Covaris, Woburn, MA, USA) and then purified by size selection with Agencourt AMPure XP beads (Beckman Coulter, Brea, CA, USA). Fragmented genomic DNA was used for library construction with the KAPA HyperPrep kits (KAPA Biosystems, Wilmington, MA, USA) per the manufacturer's protocol. Libraries were then PCR amplified and purified with AMPure XP agent (Beckman Coulter). Customized xGen lockdown probes targeting cancer‐related genes (Integrated DNA Technologies, Coralville, IA, USA) were then used for hybridization enrichment. The capture reaction was performed with Dynabeads M‐270 (Life Technologies, Carlsbad, CA, USA), xGen Lockdown hybridization, and wash kit (Integrated DNA Technologies). The enriched library was further amplified with Illumina p5 (5′ AAT GAT ACG GCG ACC ACC GA 3′) and p7 (5′ CAA GCA GAA GAC GGC ATA CGA GAT 3′) primers in KAPA Hifi HotStart ReadyMix (KAPA Biosystems) and purified with Agencourt AMPure XP beads. Libraries were quantified by qPCR with the KAPA Library Quantification Kit (KAPA Biosystems), and size distribution of each sample was examined by Bioanalyzer 2100 (Agilent Technologies, Santa Clara, CA, USA). The final libraries were sequenced on Illumina Hiseq 4000 platform for 150 bp paired‐end sequencing following manufacturer's instructions. All experimental procedures were performed using validated assays.

### 2.3 Sequencing data processing

Raw sequencing data were analyzed using a validated automation pipeline. In brief, the output BCL files (image data) from the sequencer were demultiplexed and converted into readable FASTQ files by bcl2fastq conversion (Version 1.8.4) from Illumina (San Diego, CA, USA). fastp (0.20.0) [20] was used for removing low‐quality bases (base quality score Q30 < 30), trimming adaptors, and read‐pruning. Qualified data were then mapped to the reference human genome (hg19 37d5) using Burrows‐Wheeler Aligner (bwa‐mem, v0.7.12) [21] to produce bam files. The bam files were further sorted and then filtered into the final mapped file through the process of reads deduplication, local realignment, and base quality recalibration using the sambamba (v1.3) software [22]. Local alignment around indels and recalibration of base quality score were performed with the Genome Analysis Toolkit (GATK 4.0.0) [23]. Somatic single nucleotide variations (SNVs) and insertion/deletion mutations were detected with VarScan2 [24] with the following thresholds: (1) for mutations with more than 20 recurrences in COSMIC, minimum variant allele frequency (VAF) = 0.01 with at least three minimum variant supporting reads; (2) for others, minimum VAF = 0.02 with at least five minimum variant supporting reads; (3) all variants also need to meet the standards of minimum read depth = 20, minimum base quality = 25, and variant supporting reads mapped to both strands with less than 10% strand bias. These single‐nucleotide polymorphisms (SNPs) were annotated with ANNOVAR [25] and filtered against dbSNP (v138), ClinVAR, 1000 Genome, 65 000 exomes project (ExAC), COSMIC (v70), SIFT, and in‐house database summarizing recurrent sequencing errors as previously described [19]. Gene fusions were detected by FACTERA [26] with default parameters. Copy number variation (CNV) was analyzed using ADTEx [27]. Depth ratio below 0.6 was considered CN loss (1.2 copies). Depth ratio above 2.0 (4 copies) and 1.6 (3.2 copies) were considered CN gain in tissue and plasma samples, respectively.

### 2.4 Genomic and statistical analysis

Only the gene regions covered by both panels were analyzed. A univariate Cox proportional hazard model was performed to compare survival differences to define any prognostic factors. A two‐sided *P*‐value < 0.05 was considered statistically significant. Fish plots were constructed by PyClone‐VI [28] and the timescape package [29] to track the changes in mutational profiles over time and reveal the clonal evolution of tumors. All statistical analyses and graphical illustrations were performed with r software (version 4.0.3).

## 3 Results

### 3.1 Patient characteristics

A total of 108 patients were included in the study. Fifty‐two patients received alectinib as first‐line treatment, and 56 received second‐line alectinib. The clinical features of these two cohorts are demonstrated in Table 1. All patients had discontinued alectinib treatment due to disease progression. The median time of crizotinib and alectinib treatment for all participants was 10.8 months (95% CI 8.5–14.3 m) and 7.9 months (95% CI 7.1–10.0 m), respectively. Specifically, PFS was 8.0 months (95% CI 6.9–11.1 m) for first‐line alectinib treatment and 7.9 m (95% CI 5.7–10.2 m) for second‐line treatment. Patients had confirmed *ALK* fusion variants by ARMS‐PCR or by NGS at the time of diagnosis. Of these, *EML4‐ALK* variant 1 (v1) was detected in 37 patients (34%), variant 2 in 7 patients (6%), and variant 3 (v3) in 48 patients (44%). Other *EML4‐ALK* variants and other *ALK* fusion partners were also detected in 8 patients, respectively (7%) (Table 1 and Table S1).

**Table 1:** Clinical characteristics of patients.

	First‐line alectinib	Second‐line alectinib	Total
Number of patients	52	56	108
Age			
Median (Range)	47 (26–71)	48 (25–76)	48 (25–76)
Sex, *n* (%)			
Female	33 (63%)	29 (52%)	62 (57%)
Male	19 (37%)	27 (48%)	46 (43%)
Stage, *n* (%)			
II	1 (2%)	0	1 (1%)
III	1 (2%)	2 (4%)	3 (3%)
IV	50 (96%)	54 (96%)	104 (96%)
Histology, *n* (%)			
Adenocarcinoma	50 (96%)	54 (96%)	104 (96%)
Neuroendocrine	0	2 (4%)	2 (2%)
Adenosquamous carcinoma	1 (2%)	0	1 (1%)
Pulmonary sarcomatoid carcinoma	1 (2%)	0	1 (1%)
ALK variants, *n* (%)			
*EML4‐ALK* v1 variant	14 (27%)	23 (41%)	37 (34%)
*EML4‐ALK* v2 variant	4 (8%)	3 (5%)	7 (6%)
*EML4‐ALK* v3 variant	22 (42%)	26 (46%)	48 (44%)
Other *EML4‐ALK variant*	6 (12%)	2 (4%)	8 (7%)
Non EML4‐ALK variant	6 (12%)	2 (4%)	8 (7%)
Progression‐free survival, month			
Median (95%CI)	8.0 (6.9–11.1)	7.9 (5.7–10.2)	7.9 (7.1–10.0)

### 3.2 Different landscape of genetic alterations after resistance to first‐ or second‐line alectinib

**Fig. 1:**
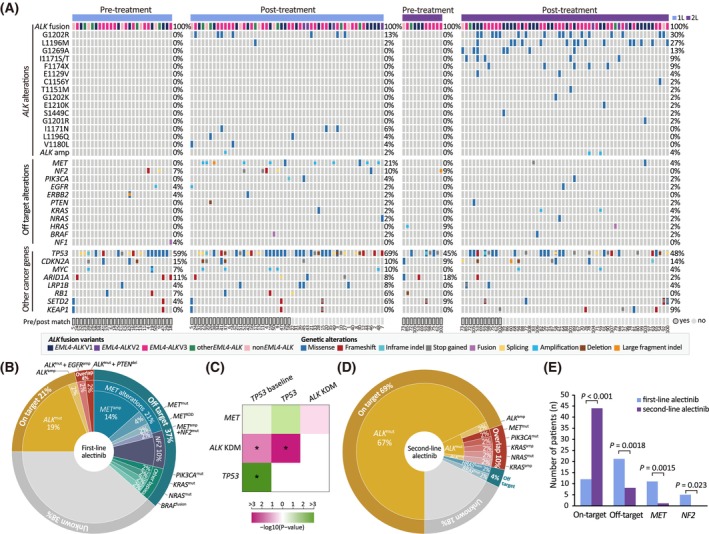
Overview of genomic alterations identified with first‐ and second‐line alectinib. (A) Genomic landscape of pre‐ and post‐alectinib samples shown in corresponding oncogenic pathways. (B) Distribution of on‐target, off‐target, and unknown resistance mechanisms in patients treated with first‐line alectinib. (C) Interaction of *TP53*, *ALK* KDM, and *MET* alterations. Green indicates co‐occurrence, and magenta indicates mutual exclusivity of alterations. Significance levels of co‐occurring or mutually exclusive events were evaluated with Fisher's exact test, with significance indicated by asterisks for *P* < 0.05. Specifically, the *P* value is 0.0017 for comparisons between *ALK* KDM and all *TP53* mutations, and 0.0087 for comparisons between *ALK* KDM and baseline *TP53* mutations. (D) Distribution of on‐target, off‐target, and unknown resistance mechanisms in patients treated with second‐line alectinib. (E) Enrichment of different resistance mechanisms after first or second‐line alectinib resistance. Significance levels are evaluated with Fisher's exact test. KDM, kinase domain mutations.

The landscape of genetic alterations in *ALK‐*positive patients treated with first‐/second‐line alectinib was illustrated in Fig. 1. On‐target alterations were detected in 13 patients post first‐line alectinib treatment (25%), including 12 with acquired *ALK* kinase domain mutations (KDM) and one with *ALK* amplification. The most prevalent KDMs were G1202R (7, 13%), I1171N (3, 6%), V1180L (2, 4%), L1196Q (2, 4%), and L1196M (1, 2%). Of these, three patients carried compound *ALK* mutations, including two with G1202R/I1171N and one with G1202R/V1180L, and two patients had concurrent off‐target mutations with *EGFR* amplification and *PTEN* deletion, respectively (Fig. 1A,B).

Off‐target alterations were defined as activating aberrations in the receptor tyrosine kinase or downstream signaling pathways other than the *ALK* gene. After first‐line alectinib treatment, known off‐target resistance mechanisms occurred in 21/52 (40%) cases, including 19 *ALK*‐independent cases (37%). Among these, *MET* was the most frequently altered gene (11, 21%), including eight *MET* amplifications (15%) and three functional mutations [L1195F, Y1248H, and kinase domain duplication (KDD)], all of which were acquired post treatment. *NF2* (5/52, 10%) was the second most common genetic alteration observed in patients receiving first‐line alectinib (Fig. 1A,B). Of these, two carried *NF2* mutations before and after alectinib treatment. Additionally, *PIK3CA* E542K, *KRAS* G12V, *NRAS* G61K, and *BRAF* fusion might also confer resistance to first‐line alectinib treatment. Importantly, the majority of these off‐target alterations were mutually exclusive with *ALK* alterations (Fig. 1B). For the 27 patients with paired pre‐ and postalectinib samples, acquired *ALK* KDMs presented significant exclusivity with baseline *TP53* mutations [Odds ratio 0.06 (95 CI, 0.0011–0.70); *P* = 0.0087, Fisher's exact test], whereas 6/7 acquired *MET* alterations had concurrent *TP53* in the baseline sample (Fig. 1C).

In patients receiving second‐line alectinib treatment, a significantly higher proportion of on‐target alterations were detected compared with that after first‐line alectinib (44/56, 79%, *P* < 0.001 Fisher's exact test) (Fig. 1D,E). These included a broad spectrum of *ALK* KDMs, including G1202R (17, 30%), L1196M (15, 27%), G1269A (7, 13%), I1171T/S (5, 9%), F1174L/V/S (5, 9%) and E1129V (2, 4%), and two *ALK* amplification (4%). Among these patients, nine carried compound *ALK* KDMs posttreatment. In contrast, a significantly lower proportion of off‐target mutations were detected post‐second‐line alectinib (7/56, *P* = 0.0018, Fisher's exact test) (Fig. 1E). Notably, only one *MET* D1228H/L1195V (co‐occurred with G1201R), but no *MET* amplification or *NF2* mutations, were found after second‐line alectinib, which was significantly lower than that after first‐line treatment (*P* = 0.0015 and 0.023, respectively, Fisher's exact test) (Fig. 1E). Other off‐target alterations included *BRAF* V600E, *HRAS* fusion, *PIK3CA* E542Q/E545K, *NRAS* G12D, and *KRAS* amplification (Fig. 1A,D). Survival outcomes from second‐line alectinib varied among patients who developed different *ALK* mutations, shortest in those with G1202R [median PFS (mPFS): 5.2 m; first‐line crizotinib, 7.3 m]. Slightly prolonged PFS for crizotinib and subsequent alectinib occurred in patients with *ALK* L1196M (mPFS: crizotinib, 10.2 m; alectinib, 8.5 m), other single *ALK* KDMs (mPFS: crizotinib, 12.4 m; alectinib, 10.4 m), or compound KDMs (mPFS: crizotinib, 9.8 m; alectinib, 9.2 m) (Fig. S2).

### 3.3 Comparing alectinib resistance mechanisms by *EML4‐ALK* variants

**Fig. 2:**
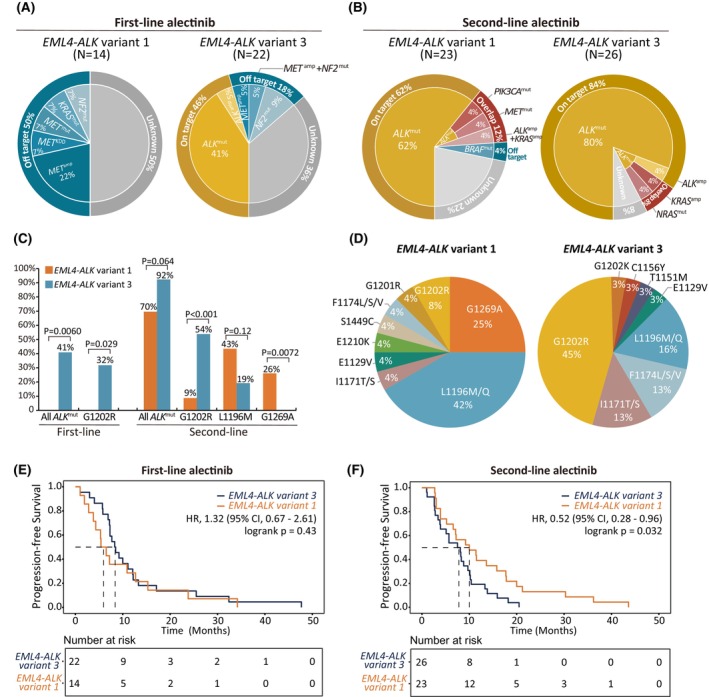
Incidence of resistance mechanisms differed by *EML4‐ALK* variants. (A) Distribution of resistance mechanisms in patients with *EML4‐ALK* v1 and v3 after first‐line alectinib. (B) Distribution of resistance mechanisms in patients with *EML4‐ALK* v1 and v3 after second‐line alectinib. (C) Enrichment of *ALK* G1202R, L1196M, and G1269A after first‐ or second‐line alectinib. Significance levels are evaluated with Fisher's exact test. (D) Prevalence of various *ALK* kinase domain mutations associated with *EML4‐ALK* v1 and v3. (E) Kaplan–Meier curves showing progression‐free survival of *EML4‐ALK* v1 and v3 patients treated with first‐line alectinib. (F) Kaplan–Meier curves showing progression‐free survival of *EML4‐ALK* v1 and v3 patients treated with second‐line alectinib. Significance levels in (E) and (F) are evaluated by the Logrank test. amp, amplification; HR, hazard ratio; mut, mutation; v1, variant 1; v3 variant3.

To explore whether *ALK* variants affect the development of different molecular mechanisms to alectinib resistance, we compared postalectinib profiles in patients with *EML4‐ALK* v1 or v3 subtypes. Interestingly, we found remarkable enrichment of off‐target alterations in v1 tumors compared to that in v3 after any line of alectinib (first‐line: v1, 50%; v3, 18%; *P* = 0.067, Fisher's exact test; second‐line: v1 16%; v3, 8%). Conversely, on‐target alterations were significantly associated with v3 (first‐line: v1, 0%; v3, 41%; *P* = 0.006; second‐line: v1 70%; v3, 92%; *P* = 0.064, Fisher's exact test) (Fig. 2A,B). Looking at individual KDMs, G1202R was markedly enriched in v3 despite the line of treatment (first‐line: v1, 0%; v3, 32%; *P* = 0.029; second‐line: v1, 9%; v3, 54%; *P* < 0.001, Fisher's exact test), indicating potential subsequent choice of third‐generation ALK TKIs. Contrarily, v1 tumors, compared to v3, exhibited more L1196M (43% vs 19%; *P* = 0.12, Fisher's exact test) and G1269A (26% vs 0%; *P* = 0.0072, Fisher's exact test) post second‐line alectinib (Fig. 2C,D). Slightly shorter PFS was observed in v1 than v3 after first‐line alectinib treatment [mPFS, 6.0 m (95%CI 4.3 m‐23.8 m) vs 8.4 m (95%CI 7.3 m‐12.2 m); HR, 1.32 (95%CI, 0.67–2.61), *P* = 0.43], probably due to the association with off‐target alterations (Fig. 2E). However, v1 patients showed significantly prolonged PFS after receiving second‐line therapy [mPFS,10.0 m (95%CI 6.6 m‐17.8 m) vs 7.8 m (95%CI 3.9 m‐10.2 m); HR, 0.52 (95% CI, 0.28–0.96), *P* = 0.032] (Fig. 2F).

### 3.4 *MET* alterations conferred early resistance to first‐line alectinib

**Fig. 3:**
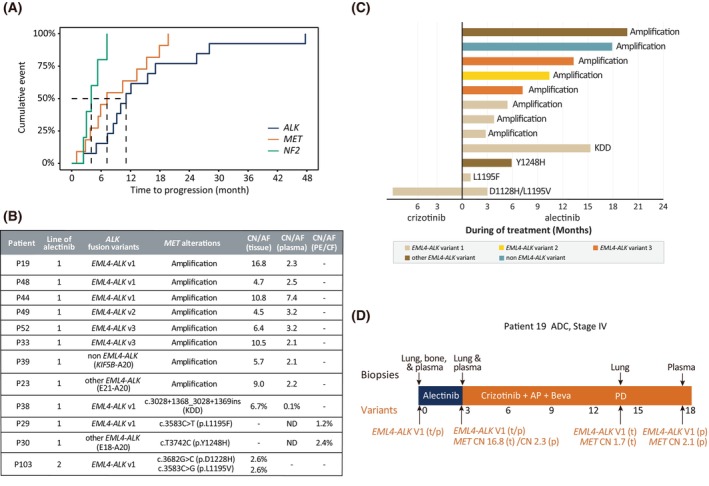
*MET* alterations conferred early resistance to first‐line alectinib. (A) Cumulative incidence of *ALK*, *MET*, and *NF2* alterations found after first‐line alectinib. (B) Copy number (CN) or allele frequencies (AF) of *MET* alterations detected in post‐alectinib tissue, plasma, pleural effusion, or cerebrospinal fluid samples of 11 patients receiving first‐line treatment and one receiving second‐line alectinib after crizotinib treatment. (C) Length of survival benefit from alectinib for different *MET* alterations and *ALK* variants. (D) Timeline of the treatments Patient 19 received. The patient was diagnosed with stage IV lung adenocarcinoma and progressed after 3 months of first‐line alectinib. *MET* amplification was detected in post‐alectinib samples. AP, cisplatin/doxorubicin; beva, bevacizumab; KDD, kinase domain duplication; p, plasma sample; PD, progressive disease; t, tissue sample.

After first‐line alectinib, off‐target alterations were more likely to confer early resistance. The median time until *MET‐*associated progression was 7.2 m (95%CI 5.4 m‐NA) with a cumulative incidence of 45.5% in the first 6 months, whereas that for *ALK* mutations was 10.0 m (95%CI 8.5 m‐25.5 m) with a cumulative incidence of 12.5% (Fig. 3A). However, 6 months following second‐line alectinib, there was a 33% incidence of developing *ALK* mutations, a rate 2.7 times higher than that after first‐line (Fig. S3A).

Overall, alectinib‐treated patients with *MET* alterations showed progression after 1.0–19.7 months, with shorter survival in *MET*‐amplified *ALK* v1 patients (Fig. 3B). *MET* amplification statuses were confirmed by tissue samples, ranging between 4.5 and 16.8 copies. However, not all plasma samples showed copy number gain (≥ 3.2 copies), reflecting possible limitations of detecting *MET* amplification using plasma (Fig. 3C). Deeper *MET* amplification could also be associated negatively with time to alectinib resistance but required further validation (Fig. S3B). Moreover, limited first‐line alectinib efficacy might also stem from concurrent genetic alterations in the primary tumor. For example, Patient 19, with baseline *RB1*, *TP53*, and *MYC* alterations, progressed after 2.8 months on alectinib and developed *MET* amplification (16.8 copies). Subsequent crizotinib (along with cisplatin/doxorubicin and bevacizumab) facilitated a 10.8‐month survival benefit and a significant reduction in *MET* (1.7 copies) (Fig. 3D), suggesting the potential of broad‐spectrum TKIs in overcoming resistance.

### 3.5 *NF2* mutations mediated primary and early resistance to alectinib

**Fig. 4:**
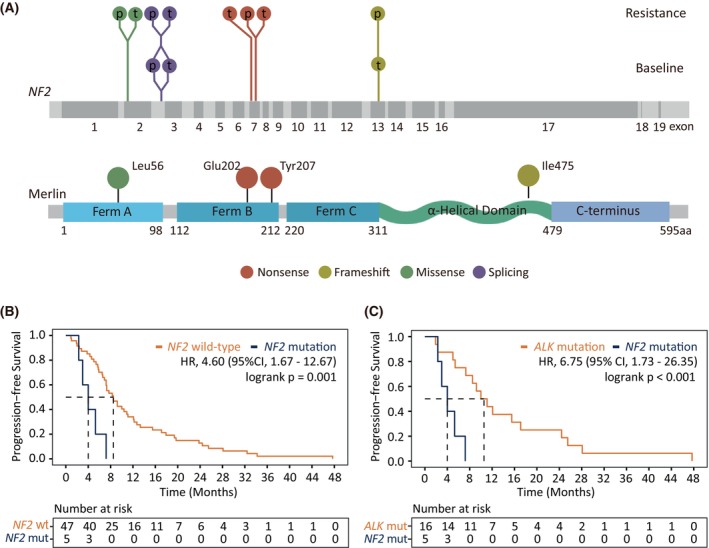
*NF2* mutations conferred early resistance to first‐line alectinib. (A) Distribution of mutations on the *NF2* gene and their corresponding positions on the Merlin protein. (B) Kaplan–Meier curves comparing progression‐free survival of patients with or without *NF2* mutations. (C) Kaplan–Meier curves comparing progression‐free survival of patients with *NF2* mutations and those with *ALK* mutations. Significance levels in (B) and (C) are evaluated by the Logrank test. HR, hazard ratio; mut, mutation; wt, wildtype.

The most prevalent off‐target alteration after 6 months of first‐line alectinib treatment was the *NF2* mutation, a tumor suppressor in the PI3K/mTOR/Akt pathway that encodes for the merlin protein (Fig. 4A). Acquired mutations included one missense mutation in exon 2 (L56R in the FERM subdomain 1) and two truncating mutations in exon 7 (E202 and Y207 in the FERM subdomain 2). Additionally, we found preexisting *NF2* mutations before any treatment, including one intron 2 splicing mutation, one exon 13 frameshift mutation (I475 in the α‐helical domain), and one large fragment deletion (Fig. 4A and Table S2). Importantly, these preexisting *NF2* mutations were associated with limited PFS after either line of alectinib treatment (P4, 3 m; P9, 2.4 m; P102, 1.1 m), indicating primary resistance (Table S2). Additionally, early development of *NF2* after alectinib also correlated with inferior outcomes, marked by a significantly shorter mPFS of 4.0 months (95%CI 3.0 m‐NA) than noncarriers (HR 4.60, 95% CI, 1.67–12.67; *P* = 0.001) (Fig. 4B). Outcomes in *NF2*‐mutation carriers were also worse compared to patients with on‐target mutations (HR 6.75, 95% CI 1.73–26.35, *P* < 0.001) (Fig. 4C), other off‐target mutations (HR 2.59, 95% CI 0.8–8.39; *P* = 0.11), and those with unknown resistance mechanisms (HR 4.29, 95% CI 1.40–13.11; *P* = 0.011) (Fig. S4).

### 3.6 Patient‐specific tumor evolution patterns

Finally, we analyzed serial tumor biopsies from *ALK*‐positive tumor treatments to understand resistance mechanisms and found three potential patterns of mutational dynamics under treatment pressure. First, continuous mutation accumulation was observed in a stage IV NSCLC patient (Patient 101), who initially responded to crizotinib and later to alectinib. Despite switching to ensartinib and lorlatinib, various *ALK* mutations such as G1202R, T1151M, and F1174V developed, indicating parallel clonal evolution leading to resistance. Secondly, Patient 73 experienced a dynamic shift of dominant mutations. Despite initially responding to crizotinib and alectinib, the predominant tumor clone containing the *ALK* G1269A mutation was overtaken by the one with G1202R. This suggested an evolution where G1202R provided a greater survival advantage, leading to tumor relapse eventually. Lastly, the development of off‐target mutations was observed in Patient 38, who had *EML4‐ALK* v1 and pleural metastasis. No secondary *ALK* mutations were found after alectinib treatment. Instead, a *MET‐KDD* mutation appeared, highlighting off‐target mutations as a resistance factor. The phylogenetic analysis showed two branches, one with suppressed *TP53* mutations and the other with the treatment‐resistant *MET‐KDD* mutation (Fig. S5).

## 4 Discussion

Alectinib has become the standard treatment for untreated *ALK*‐rearranged NSCLC patients in China since 2018. However, despite its prolonged clinical benefit in the first‐line setting, approximately 40% of patients still progressed within 2 years [2]. In this retrospective real‐world data analysis, we highlighted a group of poor responders to alectinib and explored the distinct resistance mechanisms following first‐line or second‐line treatments. By concentrating on patients who encountered early treatment failure with alectinib, we effectively enriched the specific genetic alterations that potentially contributed to resistance. This cohort was particularly intriguing to study, as these patients face unique clinical challenges that have not been fully addressed by the current standard care, compared to those who experience prolonged survival with first‐line alectinib treatment. Additionally, for better understanding, we relied on real‐world data, which captured a more diverse population than the more homogenized population in controlled clinical trials. For example, some real‐world analyses of first‐line alectinib have shown shorter PFS compared to the findings from the ALEX trial [30, 31]. Therefore, our findings may provide valuable insights for the development of alternative treatment strategies in the future.

Response dynamics and resistance mechanisms of TKI treatment largely depend on the treatment line. For many next‐generation TKIs, resistance to initial treatment is not target‐induced. For example, enhanced activities of off‐target genes and lower frequencies of *EGFR* mutations were reported after first‐line osimertinib compared to second‐line treatment [32, 33, 34, 35]. A recent investigation of the CROWN study also showed that alterations in bypass pathways, rather than single or compound *ALK* mutations, were responsible for resistance to first‐line lorlatinib [36, 37]. However, after sequential lorlatinib treatment, up to half of *ALK*‐rearranged patients developed *ALK* KDMs [38, 39, 40, 41]. Similarly, as we observed, off‐target resistance was more common after first‐line alectinib than after second‐line, predominantly from *MET* and *NF2*. *MET* activation has been a known bypass mechanism of resistance in many TKIs targeting EGFR [33, 35, 42], ROS1 [43, 44], and RET [45, 46, 47, 48]. In *EGFR*‐positive patients treated with first‐line osimertinib, *MET* amplifications typically emerged 6 months earlier than *EGFR* C797X mutations [34]. *MET* amplification was also detected in roughly 15% of *ALK*‐positive tumors after second‐generation ALK TKIs, particularly following first‐line treatment [16]. Our study and others have shown that crizotinib can be an effective sequential treatment against acquired MET amplification [13], MET‐KDD [49], activating *MET* exon 14 mutation [14] following first‐line use of second‐generation ALK TKIs. This suggests that dual *MET* and *ALK* inhibition could potentially reinstate clinical benefit. With the availability of more selective MET inhibitors, we also expect additional clinical efficacy data regarding MET‐induced second‐generation ALK TKI resistance. We also found that *MET* amplifications might be linked to *TP53* mutations in *ALK*‐rearranged patients, in line with prior reports [50, 51]. A potential mechanism for this association could involve the genetic instability of TP53‐mutated tumors, which may lead to accelerated development of resistance to targeted therapy in oncogene‐driven lung cancer. However, this mechanism requires further investigation. Given that only marginal amplification of *MET* was detected in some parallel plasma samples, our results reaffirmed the utility of tissue specimens for analyzing copy number variations and defining resistance mechanisms in the era of liquid biopsy.

Our study also identified *NF2* (neurofibromin 2) as a common off‐target gene that mediated both primary and acquired alectinib resistance. *NF2* is a tumor suppressor that encodes for the Merlin protein, which is involved in cytoskeleton stabilization and cadherin cell attachment [52]. *NF2* is a member of the canonical Hippo pathway, which inhibits proliferation and growth. NF2/Merlin also negatively regulates PI3K/mTOR/Akt signaling, and *in vitro* experiments have shown sensitivity to mTOR inhibition [53]. *NF2* inactivation is known for promoting the development of cancers in the central nervous system but is less frequent in lung cancers [54]. However, inactivated *NF2* could serve as a bypass mechanism to confer resistance to targeted therapies for lung cancers, including osimertinib, erlotinib, afatinib, and MET TKIs [33, 55, 56, 57]. Previous studies also identified *NF2* splicing site and truncating mutations following crizotinib and ceritinib treatment, and during lorlatinib treatment [58]. In our study, *NF2* mutations resulted in limited benefit from first‐line alectinib. Importantly, one FERM splicing and one a‐helical domain truncation were detected in both baseline and postalectinib specimens, suggesting *NF2*'s role in both inducing primary and secondary TKI resistance. Additionally, a baseline *NF2* large fragment insertion was not seen in subsequent plasma samples, possibly due to the inadequacy of plasma biopsies to detect large fragment alterations. Instead, *ALK* G1202R was detected. The short one‐month PFS might have resulted from the combined effect of both on‐ and off‐target resistance. For future investigations, the combination of ALK TKI with merlin re‐activation or downstream inhibition might be crucial for patients with *NF2* mutations, particularly if *NF2* is found in the baseline tumor.

Compared with off‐target alterations, our results revealed that *ALK* KDMs were later events that contributed to resistance in the first‐line setting, but interestingly not in *ALK* v1 tumors. Moreover, although previous reports have indicated an increased occurrence and variety of acquired *ALK* KDMs after second‐line alectinib [11], the spectra of mutations associated with v1 and v3 differed. Specifically, the widely recognized solvent‐front resistant mutation for second‐generation TKIs, G1202R, was particularly prevalent in v3 tumors, in concert with previous studies [8, 10, 11]. Conversely, the activation loop mutation, G1269A, and the gatekeeper mutation, L1196M, were predominantly found in v1 tumors. These two mutations together account for up to 70% of *ALK* KDMs in v1 and are frequently detected in crizotinib‐exposed tumors [10]. Biologically, *EML4‐ALK* v1 and v3 fusion proteins are different in structures and stability due to the location of EML4 breakpoints. Variant 1 breaks in the N‐terminal propeller, leading to a highly unstable product that is sensitive to Hsp90 inhibitors. In contrast, variant 3 lacks a core part of EML4's tandem atypical propeller EML (TAPE) domain, making it shorter and more structurally stable [59]. The higher stability of v3 potentially offers the cells more time to interact with alectinib and develop *ALK*‐dependent resistance, whereas a bypass pathway mutation can easily rescue v1 cells from TKI pressure. The instability of v1 might also render it more sensitive to ALK TKIs than v3 [7, 60], as is consistent with our observation in the second‐line setting. However, as no long‐term survival benefit was observed, a larger patient cohort is required to validate the effect of *EML4‐ALK* variants after first‐line alectinib. Notably, after first‐line alectinib, slightly shorter PFS was observed in v1, probably due to the early onset of off‐target alterations, including *MET* and *NF2*. Therefore, it is crucial to consider co‐mutations and response dynamics when evaluating variant‐dependent TKI efficacy and tailoring frontline therapeutic decisions in *ALK*‐rearranged patients. For example, for patients with baseline *NF2* mutations, a combination with NF2 inhibitors might be necessary to suppress *NF2*‐induced primary resistance. As very few *ALK* KDMs emerged in v1 patients after first‐line alectinib, lorlatinib could be a good alternative as the initial treatment for v1 patients. Subsequently, if v1 patients acquire *MET* amplifications upon disease progression, sequential treatment with crizotinib or lorlatinib in combination with other MET TKIs could be effective after alectinib. On the other hand, lorlatinib has shown high potency and efficacy against a broad spectrum of resistant *ALK* KDMs, including G1202R [36, 61], making it a good option for v3 patients as upfront treatment or after alectinib resistance [8]. However, upfront lorlatinib might also induce more bypass pathway mutations and currently lacks clear evidence of improving eventual overall survival [62]. Further analysis is necessary to provide more evidence for the precise sequencing of ALK TKIs.

One limitation of this study is that some patients receiving second‐line alectinib did not undergo tumor or liquid biopsy testing immediately before alectinib treatment. Consequently, some mutations detected after second‐line alectinib might have accumulated through prior crizotinib treatment. However, it is important to note that crizotinib and alectinib each demonstrate distinct resistance mechanisms. Crizotinib, as a multi‐kinase inhibitor, targets not only *ALK* but also *MET* and *ROS1*, which can lead to more off‐target effects, including mutations in the bypass genes such as *ERBB2*, *BRAF*, *KRAS*, and *FGFR3*. In fact, on‐target mutations only accounted for approximately 20%–30% of acquired mutations following crizotinib treatment, with *ALK* L1196M and G1269A being the most common [10, 63]. In contrast, alectinib is a more selective inhibitor of *ALK*‐rearranged tumors, with G1202R being a characteristic mutation associated with resistance [10]. The different spectra of acquired mutations suggest that the genomic alterations observed after second‐line alectinib were primarily the result of alectinib rather than the consequence of mutations accumulated from previous crizotinib therapy.

Other than the identified on‐ and off‐target mutations, up to 40% of patients in our study did not have a clearly defined resistance mechanism. Histological transformation is one of the reasons for TKI resistance that could not be determined through genomic analysis. For example, 15% of osimertinib‐treated patients experience phenotypic changes upon treatment failure, including squamous cell transformation or epithelial‐to‐mesenchymal transition (EMT) [33]. Such histological transformation was also evident in *ALK*‐rearranged patients and could coincide with compound *ALK* mutations [10, 40]. Therefore, future studies with posttreatment tumor biopsies are essential to better understand EMT in alectinib‐resistance patients. We also acknowledge that metastasis status and smoking history are crucial for comprehensively understanding the disease dynamics of advanced *ALK*‐rearranged NSCLC. Incomplete clinical data could be one of the factors affecting the quality of real‐world data. Future research would benefit from incorporating these critical variables to gain a more holistic view of this cohort.

## 5 Conclusions

In summary, this study illustrated the profiles of early alectinib resistance in first‐line or second‐line settings in a large cohort of alectinib‐treated patients. Off‐target *MET* and *NF2* are characteristic events associated with early resistance to first‐line alectinib, and the incidence of molecular mechanisms differed by *EML4*‐*ALK* variants. Together, our results can be helpful to guide future treatment strategies for *ALK*‐positive NSCLC patients.

## Conflict of interest

Yedan Chen, Jingwen Liu, Hairong Bao, and Yang Shao are employees of Nanjing Geneseeq Technology Inc. All other authors have no conflict of interest to declare.

## Author contributions

JH, ND, XX, YZ, HB, and YuS contributed to conceptualization; JH, ND, JL, and HB contributed to methodology; JH, ND, XX, YC, JL, and HB contributed to formal analysis; JH, ND, YC, HB, and YuS contributed to validation; JH, YZ, HB, DZ, YiS, and YuS contributed to data curation; JH, ND, XX, and YC contributed to writing—original draft; JH, ND, XX, YC, YZ, HB, YaS, and YuS contributed to writing—review and editing.

## Supporting information


**Fig. S1.** Study flowchart.
**Fig. S2.** Length of crizotinib and subsequent alectinib treatment in patients with different ALK mutations.
**Fig. S3.** MET as off target mechanism of alectinib resistance.
**Fig. S4.** Forest plots showing favorable survival of patients with or without different genetic aberrations.
**Fig. S5.** Evolutionary trajectories of three patients who developed different molecular mechanism that resulted in alectinib resistance.

**Table S1.** Clinicopathologic characteristics of all the patients enrolled in this study.

**Table S2.** Clinical and molecular features of patients with *NF2* mutations.

## Data Availability

The data that support the findings of this study are available upon request from the corresponding author at hu.jie@zs-hospital.sh.cn. The data are not publicly available due to privacy or ethical restrictions.
