# Supplementary material for: MET and NF2 alterations confer primary and early resistance to first‐line alectinib treatment in ALK‐positive non‐small‐cell lung cancer
Source: Mol Oncol. 2025 Apr 1;19(9):2715–29. doi: 10.1002/1878-0261.70029 (PMC12420365; doi:10.1002/1878-0261.70029)

***MET* and *NF2* alterations confer primary and early resistance to first-line alectinib treatment in *ALK-*positive non-small-cell lung cancer**

**Supplementary Materials**

**SUPPLEMENTARY FIGURES**

**Supplementary Figure 1. Study flowchart**

A schematic diagram showing the two cohorts of patients included in this study based on whether alectinib was used as a front-line treatment. In each cohort, the number of patients with paired samples before and after alectinib treatment, or only post-alectinib biopsies are also demonstrated.


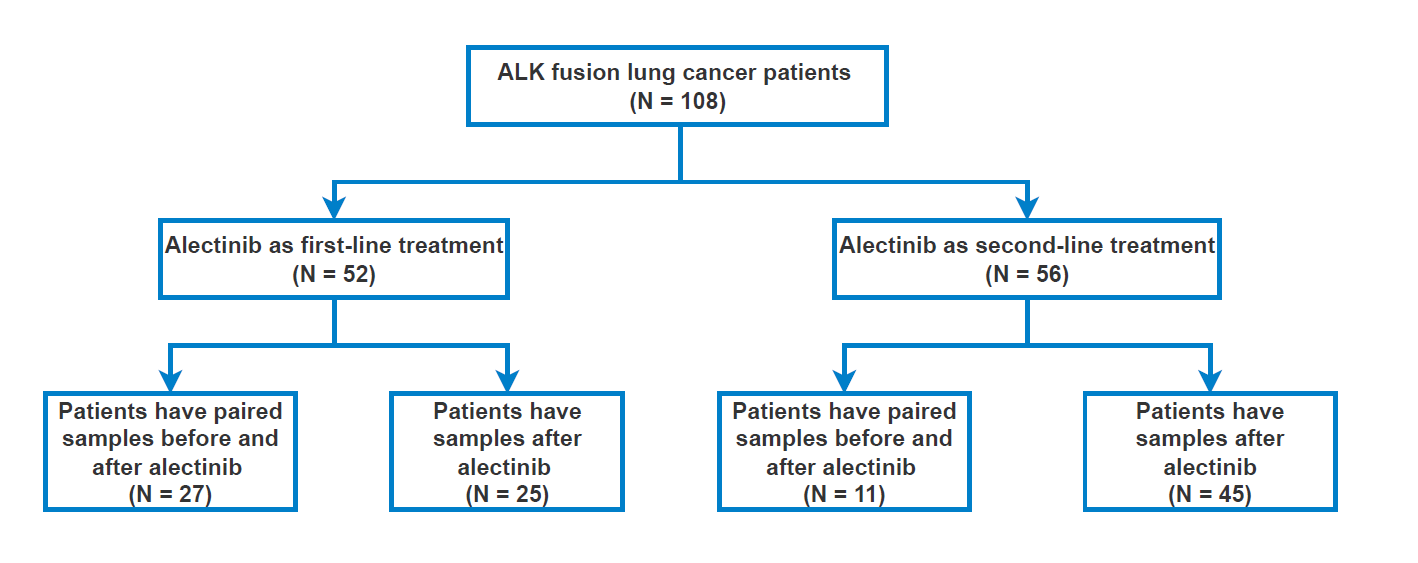


**Supplementary Figure 2. Length of crizotinib and subsequent alectinib treatment in patients with different ALK mutations.**

Patients are grouped based on the subtype of on-target mutations (*ALK* G1202R, L1196M, other point mutations, and compound *ALK* point mutations), off-target mutations, and other unknown mechanisms. Orange bars indicate the length of first-line crizotinib treatment and blue bars indicate second-line alectinib treatment these patients received.


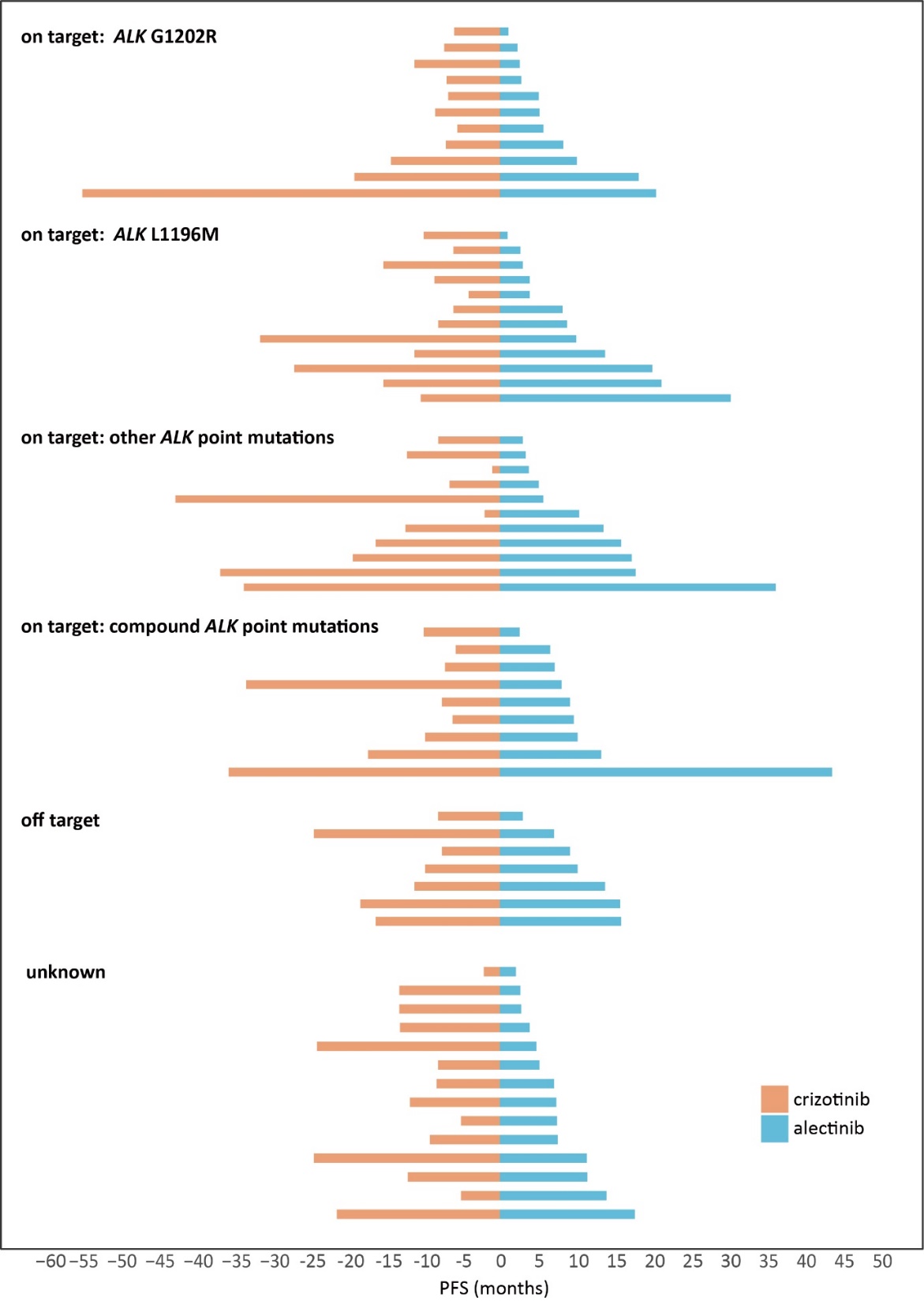


**Supplementary Figure 3. *MET* as off target mechanism of alectinib resistance.**

(a) Cumulative incidence of *ALK*, *MET,* and *NF2* alterations found with second-line treatment. (b) Relationship between progression-free survival and *MET* copy number, exhibiting a Pearson correlation coefficient of R=-0.29 with a p-value of 0.49


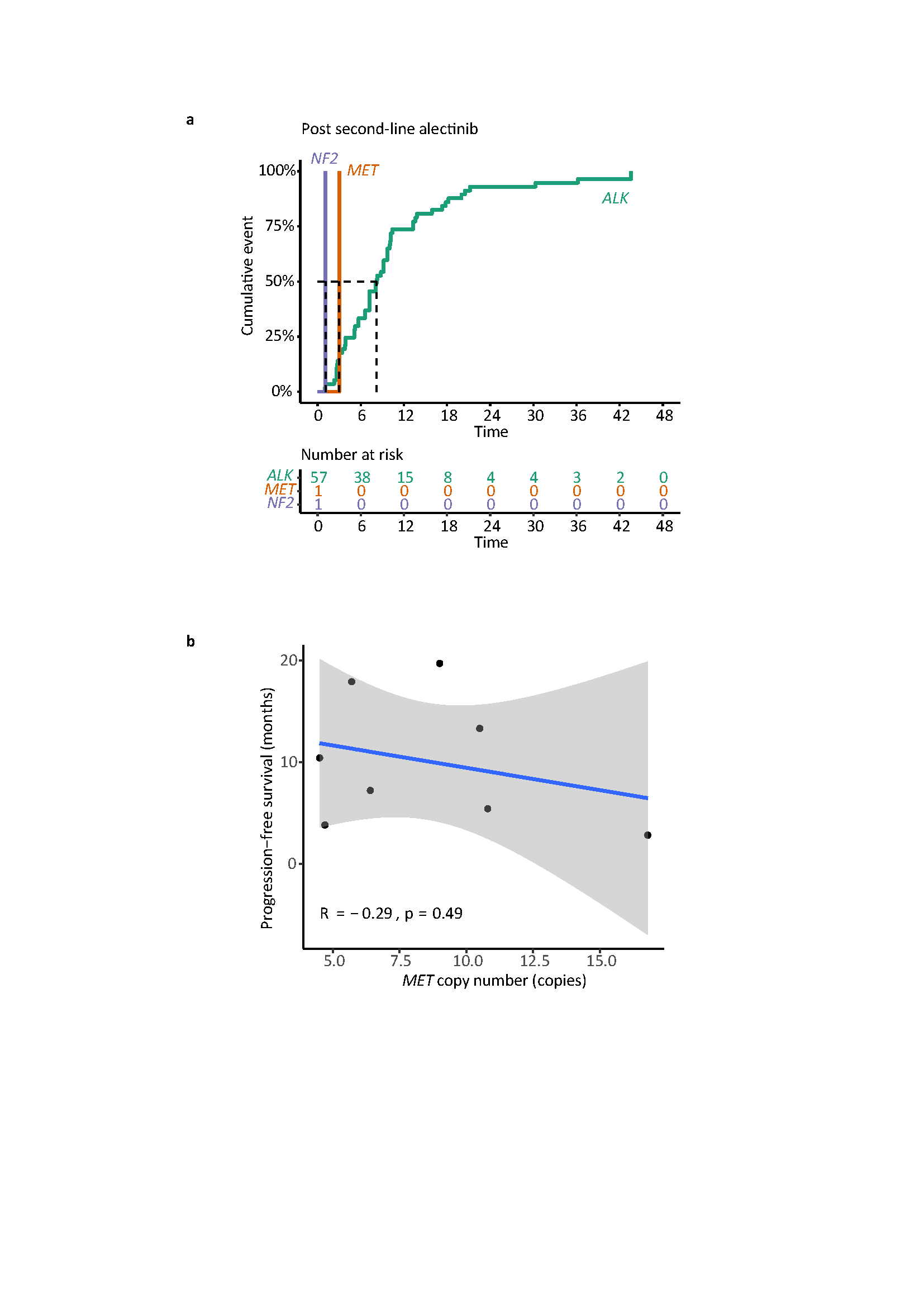


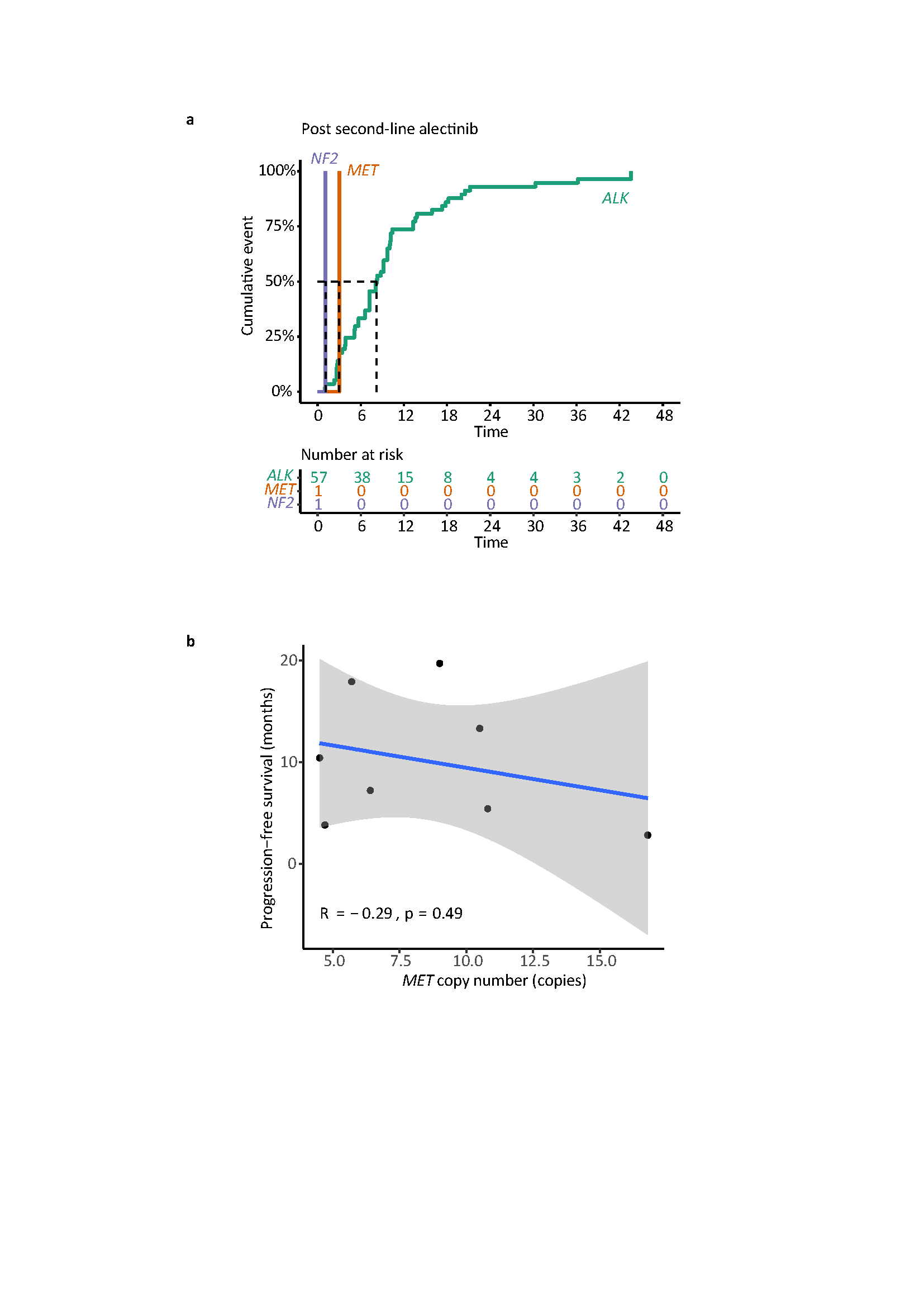


**Supplementary Figure 4. Forest plots showing favorable survival of patients with or without different genetic aberrations**

1. Comparisons among on-target alterations, *MET* alterations, and *NF2* mutations.
2. Comparisons between specific *ALK* point mutations with other on-target mutations. P values were derived using the Logrank Test. mPFS, median progression-free survival; HR, hazard ratio.


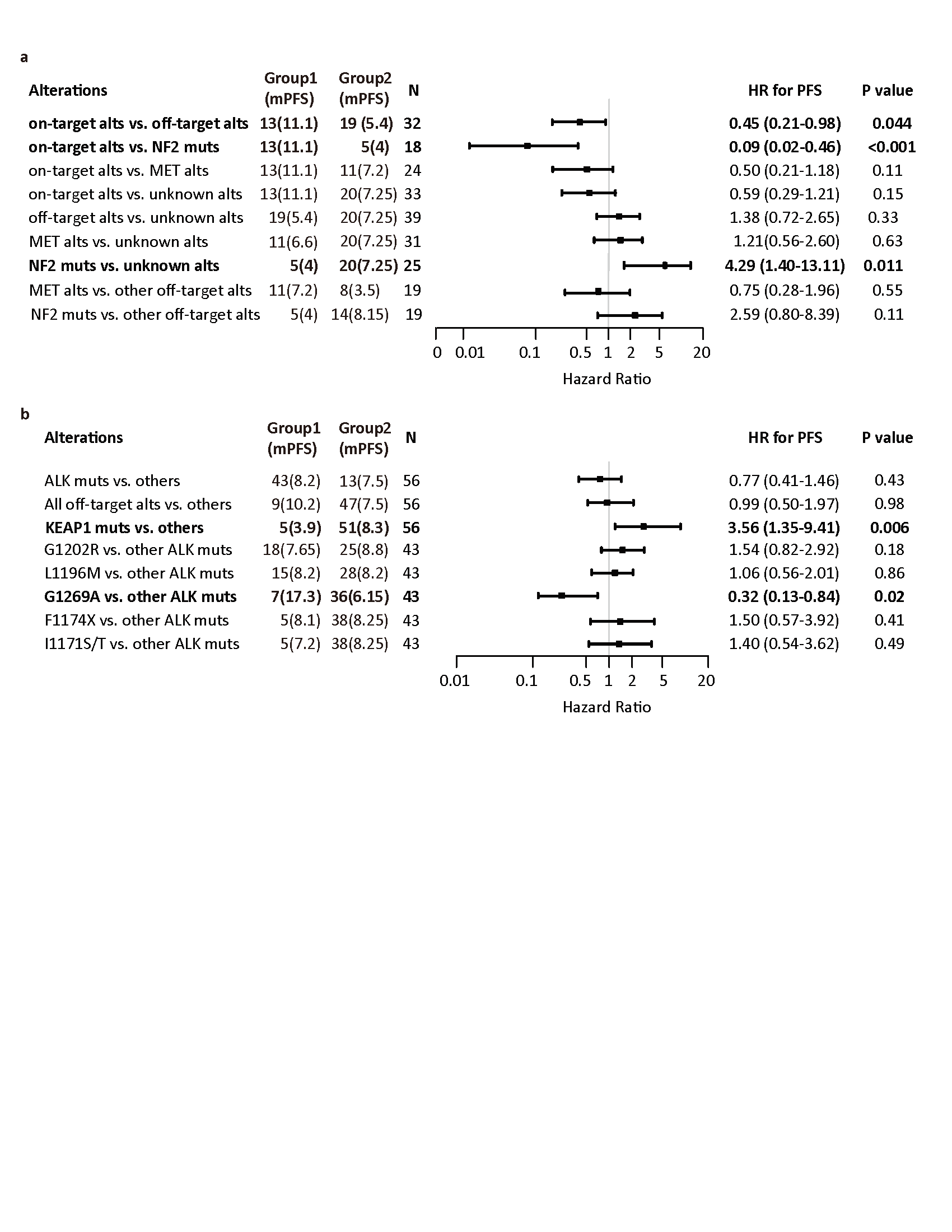


**Supplementary Figure 5. Evolutionary trajectories of three patients who developed different molecular mechanism that resulted in alectinib resistance**

1. In Patient 101, the tumor progressed through continuous accumulation of new resistant *ALK* KDMs to escape treatment pressure.
2. In Patient 73, the tumor lost the original ALK resistance mutations and acquired new on-target mutations as the tumor developed.
3. In Patient 38, off-target mutations demonstrated predominant roles in causing TKI resistance.

ADC, adenocarcinoma; PD, progressive disease; SD, stable disease


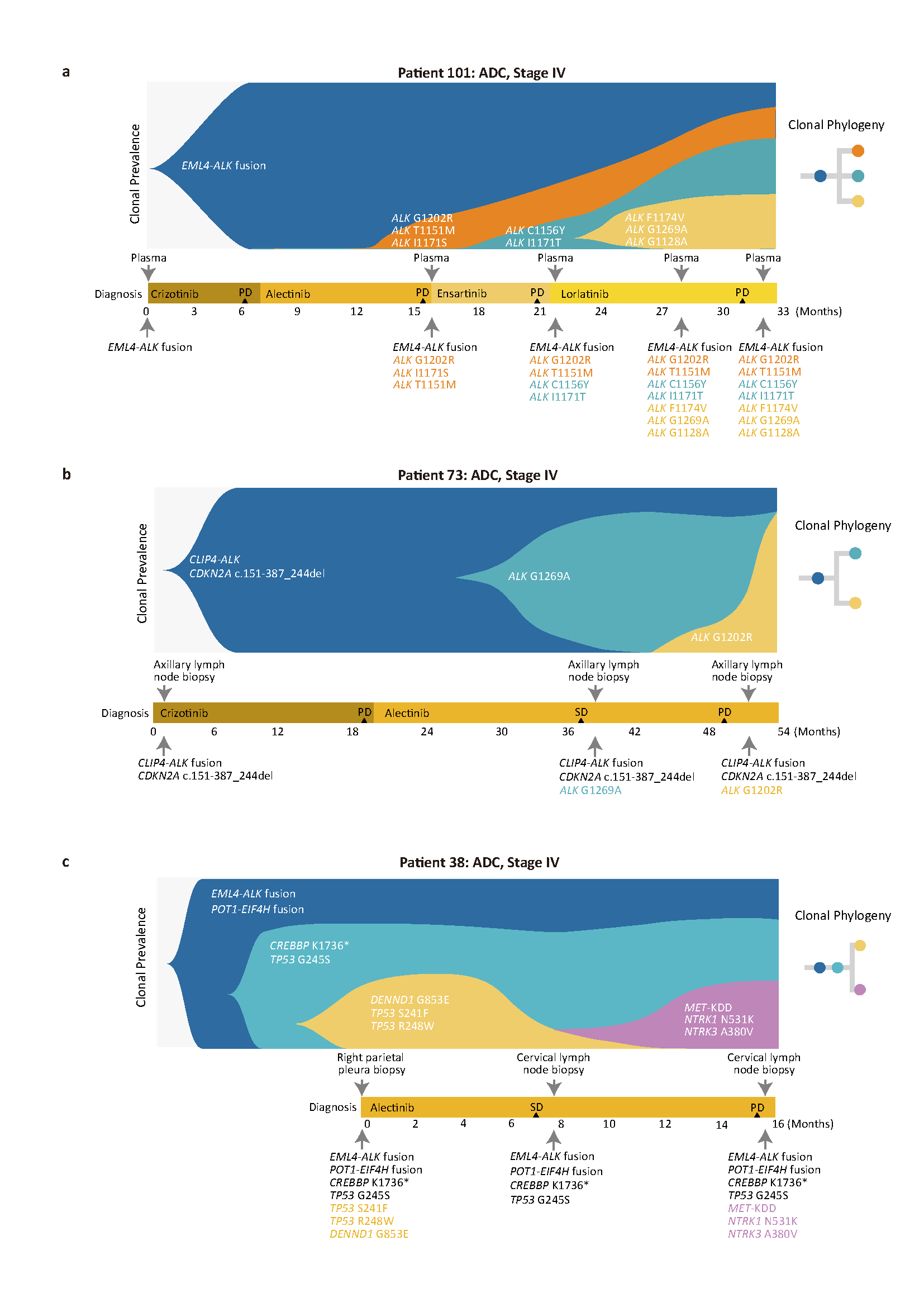

Supplement: Supplementary file 1 — Fig. S1. Study flowchart. Fig. S2. Length of crizotinib and subsequent alectinib treatment in patients with different ALK mutations. Fig. S3. MET as off target mechanism of alectinib resistance. Fig. S4. Forest plots showing favorable survival of patients with or without different genetic aberrations. Fig. S5. Evolutionary trajectories of three patients who developed different molecular mechanism that resulted in alectinib resistance. [file MOL2-19-2715-s002.docx]
